# A lurking threat: transfer of peanut allergy through peripheral blood stem cell transplantation

**DOI:** 10.1186/s40413-016-0093-4

**Published:** 2016-02-08

**Authors:** Birka Brauns, Michael P. Schön, Gerald Wulf, Martin Mempel

**Affiliations:** Department of Dermatology, Venereology and Allergology, University Medical Center, Georg August University Göttingen, Göttingen, Germany; Lower Saxony Institute of Occupational Dermatology, University Medical Center Göttingen and University of Osnabrück, Osnabrück, Germany; Department for Hematology and Oncology, University Medical Center, Georg August University, Göttingen, Germany; Dermatology Office, Elmshorn, Germany; Department of Dermatology and Venereology, University Medical Center, Rostock, Germany

**Keywords:** Peanut allergy, Stem cell transplantation, Allergy transfer, IgE-mediated hypersensitivity

## Abstract

**Background:**

There exist several reports of atopy and allergen-specific IgE-mediated hypersensitivity transferred by bone marrow transplantation, and it has been concluded that the transfer of allergic reactivity results from adoptive transfer of IgE-producing donor-derived B- and/or plasma cells. To the best of our knowledge we report the first case of peanut allergy after PBSCT.

**Case Presentation:**

A 55-year-old anciently non allergic man with secondary acute myeloid leukemia (AML) received an allogeneic peripheral blood stem cell transplantation from a matched unrelated donor following reduced-intensity conditioning. On day 32 after PBSCT, while still on prophylactic systemic immunosuppression, the patient noticed a first episode of angioedema with swelling of the nasal and oral mucosa 30 min after consuming peanut puffs. In a second episode, eight months after PBSCT, he again developed angioedema, generalized pruritus and nausea within minutes after eating biscuits containing hazelnut and peanut. Moreover, after topical application of a peanut oil-containing ointment, the patient experienced facial erythema and angioedema. Nine months after PBSCT an evaluation for peanut allergy revealed a highly increased specific IgE to peanut of 75.9 kU/l. Accordingly, skin prick tests for peanut extract were also positive. In consequence, the patient was counseled to strictly avoid peanut-related products, and provided with an emergency set. No adverse allergic events have occurred since for an observation time of 15 months after PBSCT. The stem cell donor was contacted and confirmed intolerance to peanuts. His specific serum IgE pattern nine month after PBSCT harvest was analysed and showed similar sensitization profiles compared to those of the transplant recipient.

**Conclusions:**

Because of the close temporal association between the onset of allergic symptoms in the PBSC recipient it is reasonable to assume that the acquired peanut allergy had been transferred from the donor to the recipient by the PBSC graft.

## Background

Atopy and food sensitization following solid-organ or bone marrow transplantation has previously been reported [1–6]. One theorised mechanism is the sensitization by passive transfer of donor IgE [7]. Another concept is the allergy transfer via mature specific T and/or B lymphocytes leading to active production of allergen-spezific IgE in the transplant recipient [1, 2]. The relevance of the immunosuppressive therapy (particularly of tacrolimus) for post-transplant allergies has also been previously discussed [8, 9].

Here, we present the case of a male patient who unexpectedly developed anaphylactic peanut allergy after he received peripheral blood stem cell transplantation (PBSCT) for acute myeloid leukaemia.

## Case presentation

A 55-year-old male patient who received peripheral blood stem cell transplantation (PBSCT) for acute myeloid leukaemia unexpectedly developed anaphylactic peanut allergy.

In his past medical history, the index patient had not experienced any allergic food reactions or atopy, and in particular he had regularly ingested peanut products without any problems. There was no atopic dermatitis, asthma or pollinosis in the family. The patient’s further medical history included a colon adenocarcinoma stage II treated by hemicolectomy followed by adjuvant chemotherapy in 2002 and partial nephrectomy for renal cell carcinoma stage 1a in 2006. In 2010, the diagnosis of secondary acute myeloid leukemia (AML) with NPM1-mutation and normal karyotype was established, and he received intensive induction and consolidation chemotherapy with AraC and anthracyclin-based regimens. In 2011, his AML relapsed. After achieving a second remission by salvage chemotherapy, the patient received an allogeneic peripheral blood stem cell transplantation from a matched unrelated donor following reduced-intensity conditioning. Immunosuppression for the prevention of graft-versus-host disease (GvHD) comprised pre-transplant antithymoglobulin (ATG), as well as cyclosporine (CsA) from day 0 through 120 and mycophenolate mofetil (MMF) from day 0 through 28. Hematological engraftment occurred on day 10, with complete donor chimerism in the peripheral blood achieved from day 14 on. Immune reconstitution mirrored by CD4-T-cell counts >200/μl were documented from day 120 on. The patient then developed extensive chronic GvHD of the skin, necessitating local and systemic steroids as well as tacrolimus at trough levels (5–10 ng/ml) starting at 6 months after transplantation.

On day 32 after PBSCT, while still on prophylactic systemic immunosuppression and receiving an otherwise defined low-bacteria lactose-free diet, the patient noticed a first episode of angioedema with swelling of the nasal and oral mucosa 30 min after consuming peanut puffs. In a second episode, eight months after PBSCT while on normal nutrition, he again developed angioedema, generalized pruritus and nausea within minutes after eating biscuits containing hazelnut and peanut. There have been no cardiovascular symptoms. The patient was successfully treated intravenous with 250 mg methylprednisolone and 2 mg clemastine.

Moreover, between 2^nd^ and 4^th^ week after PBSCT occasional topical application of a refined peanut oil-containing lipid replenishing cream induced skin erythema and pruritus within minutes.

These events prompted an evaluation for peanut allergy nine months after PBSCT, revealing a total IgE of 202 kU/l and a highly increased specific IgE to peanut of 75.9 kU/l. In detail, we found high specific IgE to the major recombinant peanut allergens Ara h1, Ara h2 and Ara h3 (Table 1). Accordingly, skin prick tests for peanut extract were also positive (>6 mm). In consequence, the patient was counseled to strictly avoid peanut-related products, and provided with an emergency set (containing self-injectable epinephrine, liquid oral antihistamines and steroid). The patient strictly avoided further exposition to peanut products, and no further adverse allergic events occurred. Unfortunately, the patient succumbed to progressive leukemia 36 months after PBSCT.

**Table 1:** Specific IgE and skin prick test in the transplant recipient and the transplant donor

Test	Allergen	Results 9 months after PBSCT	
		Transplant recipient	Transplant donor
sIgE	Total IgE	202 kU/l	361 kU/l
	Peanut	75.9 kU/l	>100 kU/l
	rArah1	7.02 kU/l	45.3 kU/l
	rArah2	7.73 kU/l	30.9 kU/l
	rArah3	36.50 kU/l	31.70 kU/l
	rArah8	<0.35 kU/l	0.68 kU/l
	rArah9	<0.35 kU/l	<0.35 kU/l
Skin prick test	Peanut (1:10)	++++ (>6 mm)	Not performed

*sIgE* serum IgE, *PBSCT* peripheral blood stem cell transplantation

The stem cell donor was contacted and confirmed clinical allergy to peanuts and negated any other allergies. His specific serum IgE pattern nine month after PBSCT harvest was analysed and showed similar sensitization profiles compared to those of the transplant recipient with increased specific IgE to peanut and high specific IgE to the major recombinant peanut allergens Ara h1, Ara h2 and Ara h3 (Table 1).

Although the sensitization profiles of donor and recipient (reactions against Ara h1, Ara h2 and Ara h3) can be found in a large proportion of true peanut allergic patients it is reasonable to assume that the acquired peanut allergy had been transferred from the donor to the recipient by the PBSC graft.

There exist several reports of atopy and allergen-specific IgE-mediated hypersensitivity other than peanut transferred by bone marrow transplantation, and it has been concluded that the transfer of allergic reactivity results from adoptive transfer of IgE-producing donor-derived B- and/or plasma cells [1, 2]. The case of the index patient presented here is compatible with this concept of allergy transfer via mature specific memory B-cells, eliciting a reactivity pattern almost identical to that of the donor. To the best of our knowledge it is the first case of peanut allergy after PBSCT. Numbers of transplanted cells were generally higher in PBSC than BM grafts (e. g. G-CSF-primed peripheral blood grafts contain approximately 10-fold more T-cells) and are associated with better engraftment but increased risk of (chronic) GvHD. The mature specific memory B-cells in the index patient must have been either circulating blood B-cells or they had been mobilized from the marrow into the blood by the G-CSF pre-treatment of the PBSC donor. An additional pro-allergenic influence of progenitor cell mobilization with G-CSF is conceivable.

In addition to the late reaction to peanut products attributable to the engraftment of specific B-cells, the index patient had also experienced mild reactions to peanut products between 2^nd^ and 4^th^ week after transplantation. While assuming that this reaction already was a symptom of the peanut reactivity, it appears unlikely that the B-cells transferred with the graft might have produced the amounts of IgE responsible for the reaction at that early time point. Yet, because the graft had been transplanted in a volume of 300 ml, it is conceivable that passive transfer of IgE itself in the PBSC-bag or of cell-bound IgE contributed to the reaction. Such passive and transient transfer of peanut allergy has been described in solid-organ transplants for patients having received liver [3], lung [4], combined liver-kidney transplants [5] or combined pancreas-kidney transplants [6]. While the transfer of IgE-mediated allergy in BMT can be explained by the transfer of long-living plasma cells in the bone marrow [10], solid organs such as the lungs have not been described to harbor such long-living plasma cells. Alternatively, IgE itself has been described a carrier of IgE-mediated memory [7] and might therefore be responsible for persisting allergic reactions.

The relevance of the immunosuppressive therapy with CsA, MMF and tacrolimus for post-transplant allergies was previously discussed. In particular tacrolimus seems to be a potential risk factor, as it may lead to a Th1/Th2 imbalance towards Th2 and also inhibited the regulatory T cells by suppression of interleukin 2 [8, 9]. Thus in our case an additional effect of tacrolimus on the occurrence of posttransplant peanut sensitization appears conceivable. Especially in patients with altered skin barrier function the cutaneous exposure to food protein can induce allergic sensitization [11]. Refined topical peanut oil products as applied by our patient are considered to be safe and the risk to induce type I allergy very low [12]. However an additional effect with sensitization via the peanut containing ointment is therefore unlikely in our case in our opinion, but might principally also be possible.

## Conclusion

This case documents a probable stable transfer of IgE-memory cells by peripheral stem cell transplantation, which elicitied significant clinical symptoms. Although uncommon, the consequences of transplant-conveyed allergies can be life-threateningly severe. Thus, exclusion of significant type-I allergies prior to performing PBSCT appears to be a worthwhile investment. In case of known hypersensitivity in the donor, we recommend adequate testing and counselling of the PBSC recipient.

## Consent

Written informed consent was obtained from the patient for publication of this Case Report and any accompanying images. A copy of the written consent is available for review by the Editor-in-Chief of this journal.

## Abbreviations

BMT: bone marrow transplantation
cGvHD: chronic graft versus host disease
CsA: Cyclosporine
MMF: mycophenolate mofetil
PBSCT: peripheral blood stem cell transplantation
sIGE: serum IGE
